# Influenza viruses and coronaviruses: Knowns, unknowns, and common research challenges

**DOI:** 10.1371/journal.ppat.1010106

**Published:** 2021-12-30

**Authors:** Olivier Terrier, Mustapha Si-Tahar, Mariette Ducatez, Christophe Chevalier, Andrés Pizzorno, Ronan Le Goffic, Thibaut Crépin, Gaëlle Simon, Nadia Naffakh

**Affiliations:** 1 CNRS GDR2073 ResaFlu, Groupement de Recherche sur les Virus Influenza, France; 2 CIRI, Centre International de Recherche en Infectiologie (Team VirPath), Inserm U1111, Université Claude Bernard Lyon 1, CNRS UMR5308, ENS de Lyon, Lyon, France; 3 Inserm U1100, Research Center for Respiratory Diseases (CEPR), Université de Tours, Tours, France; 4 IHAP, UMR1225, Université de Toulouse, ENVT, INRAE, Toulouse, France; 5 Université Paris-Saclay, UVSQ, INRAE, VIM, Equipe Virus Influenza, Jouy-en-Josas, France; 6 Institut de Biologie Structurale (IBS), Université Grenoble Alpes, CEA, CNRS, Grenoble, France; 7 Swine Virology Immunology Unit, Ploufragan-Plouzané-Niort Laboratory, ANSES, Ploufragan, France; 8 RNA Biology and Influenza Virus Unit, Institut Pasteur, CNRS UMR3569, Université de Paris, Paris, France; University of Pittsburgh, UNITED STATES

## Abstract

The development of safe and effective vaccines in a record time after the emergence of the Severe Acute Respiratory Syndrome Coronavirus 2 (SARS-CoV-2) is a remarkable achievement, partly based on the experience gained from multiple viral outbreaks in the past decades. However, the Coronavirus Disease 2019 (COVID-19) crisis also revealed weaknesses in the global pandemic response and large gaps that remain in our knowledge of the biology of coronaviruses (CoVs) and influenza viruses, the 2 major respiratory viruses with pandemic potential. Here, we review current knowns and unknowns of influenza viruses and CoVs, and we highlight common research challenges they pose in 3 areas: the mechanisms of viral emergence and adaptation to humans, the physiological and molecular determinants of disease severity, and the development of control strategies. We outline multidisciplinary approaches and technological innovations that need to be harnessed in order to improve preparedeness to the next pandemic.

## Introduction

The years 2020 to 2021 are characterized by an outstanding and worldwide research effort aimed at mitigating the Coronavirus Disease 2019 (COVID-19) pandemic, leading to >145,000 published articles and >2,900 completed or underway clinical trials (https://www.covid-trials.org). Experience and research work related to previous outbreaks, including the emergence of the H1N1pdm09 influenza virus in 2009, was also leveraged. As a result, safe and effective vaccines [1] as well as monoclonal antibody–based treatments [2] have been developed in less than 1 year. At the same time, the COVID-19 crisis exposed the weaknesses of our preparedness and response to pandemics and highlighted large gaps that remain in our knowledge of the biology of coronaviruses (CoVs) and influenza A viruses (IAVs), the 2 major zoonotic respiratory viruses with pandemic potential. Here, we sought to identify common challenges of influenza virus and CoV research that should be addressed in order to become better prepared for upcoming pandemics.

## 1. Emergence, transmission, and adaptation to humans

### 1.1. Mapping of animal reservoirs and intermediate hosts

Wild waterfowl are the main reservoir for IAVs, with poultry and swine being evolutionary intermediaries and possibly “mixing vessels” for the transmission to humans [3]. Human CoVs come from wild animal reservoirs as well, especially bats as in the case of Severe Acute Respiratory Syndrome Coronavirus 2 (SARS-CoV-2) or rodents [4,5]. They are thought to emerge in humans through an intermediate mammalian host, possibly domesticated or tamed or hunted, e.g., dromedary camels for Middle East Respiratory Syndrome Coronavirus (MERS-CoV) and civets for Severe Acute Respiratory Syndrome Coronavirus (SARS-CoV) [4]. However, the direct animal progenitor of SARS-CoV-2 remains elusive to date [6].

Neither the emergence of H1N1pdm09 virus nor that of SARS-CoV-2, the viruses responsible for the 2 last pandemics (**Table 1**), were preceded by a disease outbreak in an animal population. This fits with phylogenetic data suggesting that viruses potentially “preadapted” to humans could have evolved and circulated undetected in wild or domesticated animals for years [7,8]. This situation, along with the genetic plasticity of IAVs and CoVs, as well as the diversity of animal species that could potentially represent prepandemic reservoirs, makes it an unrealistic goal to identify all viruses with pandemic potential before they emerge in humans.

**Table 1:** Basic features of H1N1pdm09 and SARS-CoV-2 viruses.

	H1N1pdm09	SARS-CoV-2
Family	Orthomyxoviridae	Coronaviridae
Genus	Alphainfluenzavirus	Betacoronavirus
Subgenus or subtype Surface glycoproteins	H1N1 HA NA	Sarbecovirus S
Genome	Single-stranded negative sense RNA, segmented	Single-stranded positive sense RNA, linear
Genome size	13.5 kb	29.9 kb
Evolution processes	Genetic drift (mutations) and shift (reassortments)	Genetic drift (mutations) and shift (recombinations)

HA, hemagglutinin; NA, neuraminidase; S, Spike; SARS-CoV-2, Severe Acute Respiratory Syndrome Coronavirus 2; H1N1pdm09, H1N1 pandemic 2009.

However, once a cluster of human zoonotic cases has been detected, the capacity to rapidly characterize the animal origin, route of transmission, and site of emergence of the pathogen is essential for informed public health decisions and early control of the outbreak. Such a capacity requires extensive and long-term surveillance data sets on the spatial and temporal dynamics of viruses in their reservoir and intermediate host species. With regard to IAVs, major progress has been achieved in recent years. Tracking of the epidemiology and evolution of highy pathogenic avian IAVs has improved due to the rise in whole genome sequencing [9] and initiatives on sharing sequencing data such as the GISAID database [10]. Genomic surveillance was integrated with the collaborative expertise of virologists, ornithologists, ecologists, and mathematical modelers to identify bird species, time periods, habitats, and geographies that are associated with increased risks of transmission to humans and therefore require an active surveillance of wild and domestic animals [11,12]. This approach should be further developed and extended to CoVs. Zooanthroponosis, as exemplified by the transmission of the H1N1pdm09 and SARS-CoV-2 human viruses back to animal species [13,14], should be closely monitored. Indeed, such spillover events can lead to the selection of variants at the time of cross-species transmission, such as SARS-CoV-2 variants isolated in mink farms from the Netherlands and Denmark, which showed amino acid substitutions in the spike protein possibly increasing its affinity for the mink angiotensin converting enzyme 2 (ACE2) receptor [15], or to the accumulation and fixation of mutations over time, as observed upon introduction of the human H1N1pdm09 virus in swine [16]. Ultimately, these events can lead to the establishment of novel animal virus lineages with subsequent risks for animal and human health [17]. To this end, taking advantage of recent developments in next generation sequencing technologies, such as the Oxford Nanopore MinION, in-field collection of genomic and metagenomic data should be intensified and combined with complementary approaches such as syndromic surveillance and serological surveys in farmed species [18–20].

Finally, research aiming at further understanding the molecular determinants for the host range and host switching potential of IAVs and CoVs will help identify high-risk viruses. This knowledge, together with surveillance data, should ultimately serve to feed and refine the risk assessment tools that have already been implemented for IAVs, such as the World Health Organization’s (WHO) Tool for Influenza Pandemic Risk Assessment (TIPRA) and the Centers for Disease Control and Prevention’s (CDC) Influenza Risk Assessment Tool (IRAT) [21], and adapt them to CoVs.

### 1.2. Understanding how selective pressures are shaping viral evolution

IAVs and CoVs harbor single-stranded RNA genomes (negative or positive sense, respectively). High mutation rates occur during replication, which allows them to evolve rapidly [22,23]. There is much evidence that CoVs can limit their mutation rates due to a proofreading mechanism of their polymerase [24,25]. However, it is unclear to what extent this proofreading mechanism is limiting CoVs diversification in an epidemiological context [23,26]. Both viral families also undergo evolutionary shortcuts (reassortment of genomic segments for IAVs and homologous recombination for CoVs), which may favor the emergence of pandemic viruses. For instance, the H1N1pdm09 virus was a complex reassortant harboring genomic segments of swine, avian, and human IAVs origin [7]. SARS-CoV-2 is characterized by a polybasic cleavage site in the spike glycoprotein that was possibly acquired from another bat CoV through recombination [27]. Interestingly, recurrent occurrence of short deletions are currently being observed in the spike glycoprotein of SARS-CoV-2 [28,29] and in the hemagglutinin glycoprotein of H1N2 swine IAVs [30,31].

Depending on the nature of the host barrier and the level of preexisting immunity in the human population, CoVs or IAVs transmitted from animals to humans can show different patterns of pathogenicity and human-to-human transmissibility, which will result in different evolutionary pressures acting on the viruses. For instance, the emerging SARS-CoV-2 and H1N1pdm09 IAV were both less deadly and more transmissible among humans than the SARS-CoV, MERS-CoV, and zoonotic H5N1/H7N9 IAVs, but SARS-CoV-2 and the 2009 influenza pandemic virus differed in that the mortality was the highest in people older than 70 years for the former but not the latter [32]. Improving the accuracy of phylogenetic methods that infer the evolutionary history of the emerging viruses by sequence comparison with samples derived from the reservoir and intermediate hosts (see Section 1.1) will help characterize the pattern of host species jump. For the H1N1pdm09 as well as the SARS-CoV-2 viruses, real-time monitoring of the emerging virus evolution has been a key component of the pandemic response, not only to track transmission chains and evaluate the reproductive number [33,34] but also to infer the spatiotemporal spread of the virus country-wide or worldwide and evaluate the efficacy of mitigation measures [35,36]. A major challenge is to ensure the representativeness of sequences and to improve the predictive value of data-driven mathematical models so that they accurately anticipate how the course of the pandemic will be affected by the rise of new viral variants and by the implementation of mitigation measures [37,38]. Another challenge in the field is to combine phylogenetic and phenotypic data (e.g., virulence, transmissibility, escape from the antibody or T-cell response), in order to better understand how selective pressures and trade-offs are shaping viral evolution at both the intra- and interhost levels. For instance, the often mentioned hypothesis that virulence and transmissibility are inversely correlated and that this trade-off determines evolutionary trajectories remain to be verified for emerging IAVs and CoVs [39]. While the H1N1pmd09 remained genetically stable several years after its emergence, it has taken less than 1 year to see SARS-CoV-2 variants with distinct phenotypes from the progeny virus emerge, possibly due to differences in the nature and effects of cross-immunity. An hypothesis is that some early SARS-CoV-2 variants may have emerged from immunocompromised patients with long-lasting infection and, possibly, treatment with plasma from convalescent patients or recombinant antibodies [40]. Which mutations are evolutionary neutral or are associated with immune escape, increased replication, or increased transmissibility remains to be fully evaluated. Mutations on the viral spike protein are best characterized so far. Notably, the N501Y mutation, shared by the 4 main variants of concern (B.1.1.7, P.1, B.1.351 and B.1.1.529), increases the spike’s affinity of the ACE2 receptor and could thereby increase transmissibility, whereas the E484K mutation, found in the P.1 and B.1.351 variants, decreases binding of neutralizing antibodies and could thereby favor immune escape (for a review, see [41,42]). Unraveling the evolutionary drivers of IAVs and CoVs genetic diversity will help improve policy responses as well as the design of vaccines and antiviral therapies in the future.

### 1.3. Investigating the mechanisms of transmission through aerosols

There is now much evidence for mid- to long-range (>2 m) airborne and direct contact transmission of IAVs and CoVs [43,44]. Indirect contact transmission via contaminated surfaces or objects, also called fomites, can occur according to environmental sampling data [43]. However, it remains unclear how efficiently it does occur in real-life situations [45,46]. By contrast, the contribution of virus aerosolization to viral dissemination in the human population has been largely documented mostly in indoor environments (e.g., [47,48]) and is considered less likely in outdoor conditions [49]. The contribution of aerosols to the bird-to-bird spread of highly pathogenic avian IAVs in poultry farms, with serious consequences for the poultry industry, is also of great concern [50,51].

**Fig 1:**
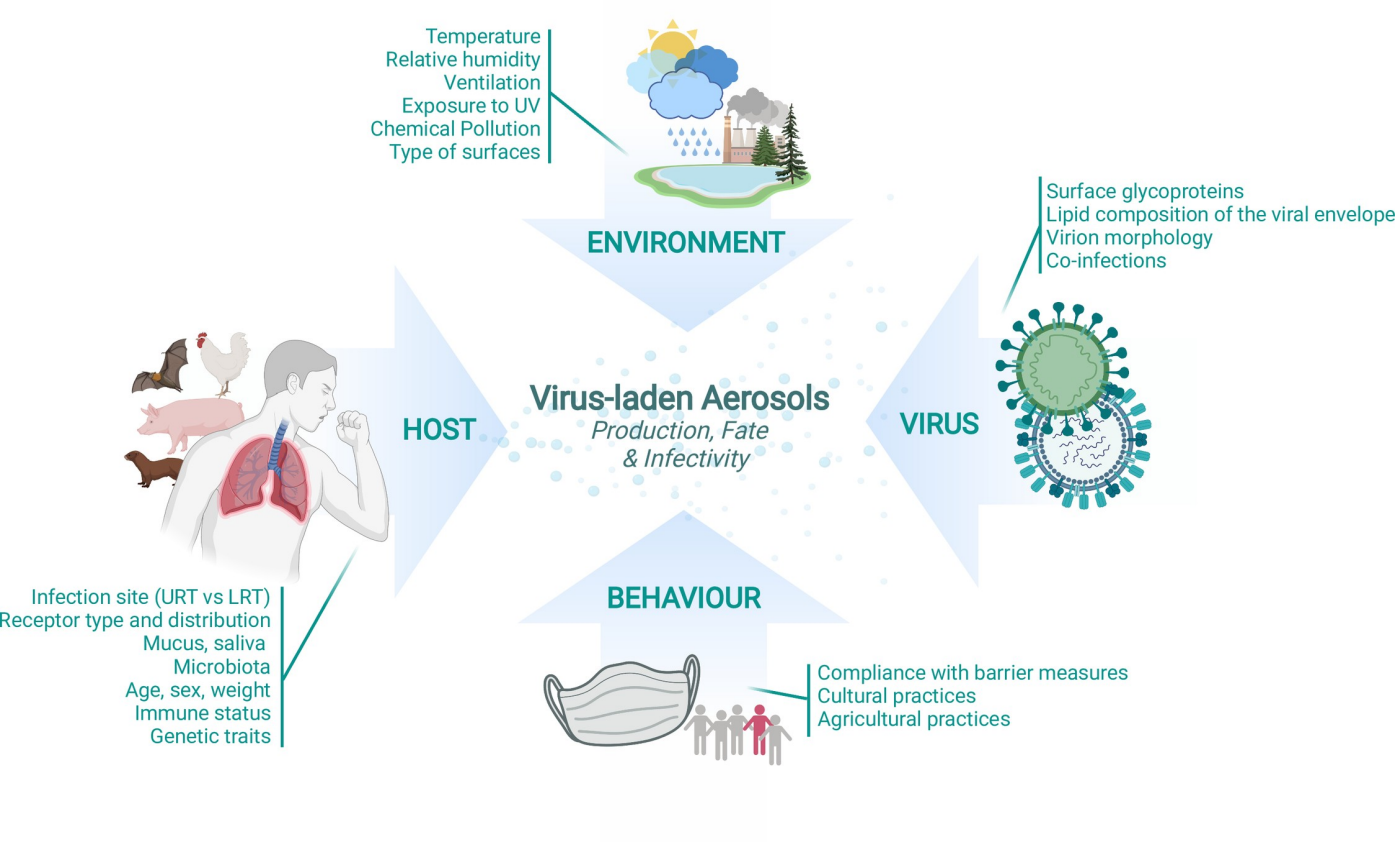
Factors potentially affecting the production, fate, and infectivity of virus-laden aerosols. Only few (mostly environmental and viral factors) have been documented so far. Multidisciplinary progress in this research field will help improve mitigation measures when a new pandemic respiratory virus emerges. Created with BioRender.com. LRT, lower respiratory tract; URT, upper respiratory tract; UV, ultraviolet.

Transmission via aerosols remains poorly understood and largely understudied. A traditional distinction is made between large respiratory virus-containing droplets (>5 μm) that usually fall to the ground within 2 meters and viral aerosols formed of droplets <5 μm in size, which can be transported in the air up to 70 meters [43]. This distinction has been recently challenged, as (i) droplets generated upon coughing, sneezing, talking, and breathing span a continuous range of size from 0.01 to hundreds of microns [52]; and (ii) their size can vary over time through evaporation, along with their composition and morphology [53]. There is an urgent need to investigate the processes of aerosols production, dispersion in the air, deposition on surfaces and decay, and to understand how the production, fate, and infectivity of aerosols are affected by environmental, biological, and behavioral parameters (**Fig 1**). Studies on the effect of temperature and relative humidity [54,55] should be pursued and extended to other environmental parameters such as exposure to UV, chemical pollution, and ventilation. In addition, methodological advances are needed regarding aerosol sampling and monitoring [56,57], as well as aerosol reduction and inactivation. Finally, understanding what determines the extent to which asymptomatic individuals can transmit respiratory viruses will be essential to guide nonpharmaceutical interventions and vaccination strategies. It was established as of spring 2020 that asymptomatic SARS-CoV-2 carriers could transmit the virus [58,59]. This question has long been understudied for influenza viruses; however, there is a recent report that asymptomatic individuals can transmit seasonal influenza viruses to approximately 6% of household contacts [60].

The environmental persistence and dissemination of IAVs have been shown to also depend upon viral and host determinants [43,61]. Traits of the hemagglutinin surface protein that increase receptor binding were found to increase the efficiency of IAV transmission between mammals [62,63]. Similarly, changes in the spike surface protein of the SARS-CoV-2 that increase receptor binding confer a higher human-to-human transmissiblity [64]. Mutations resulting in an increased stability of the hemagglutinin were also found to increase the infectivity of viruses isolated from air exhaled by infected ferrets [65] and to increase ferret-to-ferret transmission via the aerosol route [62]. The impact of other viral features, such as the morphology of IAV and CoV particles, on aerosol transmission remains to be documented. Regarding host factors, basic questions remain unanswered such as the impact of age (children versus adults) and site of infection (upper or lower respiratory tract) on the characteristics of aerosols in terms of size distribution, chemical composition, mucus/saliva/cell content, and infectivity of the droplets. Investigations of superspreading events suggested that the nasal microbiome and respiratory coinfections could influence the airborne transmission of IAVs and SARS-CoV-2, which deserves further studies [66,67]. Age and obesity were found to be associated with an increased release of SARS-CoV-2–laden aerosols [68] and could possibly contribute to superspreading events along with other host and environmental conditions [66]. Epidemiological and viral shedding data suggest that obesity may play a role in influenza transmission [69], a question that could be investigated further using the recently developed obese ferret model [70]. There is evidence that host genetics can determine the susceptibility to severe influenza [71] and COVID-19 [72,73]. However, evidence is so far lacking for a role of host genetics in determining the route and propensity of transmission of respiratory viruses.

## 2. Determinants of viral load dynamics and disease severity

### 2.1. Deciphering how the immune and inflammatory responses contribute to severe pathogenesis

In severe influenza and COVID-19 cases, elevated systemic levels of interferons, cytokines, chemokines, and other inflammatory mediators are a major cause for fatal outcome [1,74,75]. They are usually associated with acute mononuclear/neutrophilic inflammatory infiltration in the lower respiratory tract and with diffuse alveolar damage, which impair gas exchange and blood oxygenation. In addition, patients with severe COVID-19 show vascular damage and thrombosis according to autopsy findings [76] and dysregulated function of T cells [77,78]. Uncovering the multiple and complex mechanisms that control the innate and adaptative responses to viral infection is paramount for the design of effective and safe immunomodulatory therapies (see Section 3.1). The hyperinflammatory profiles of influenza and COVID-19 patients show similarities, e.g., high serum levels of interleukin (IL)-6 and tumor necrosis factor (TNF) alpha, and also exhibit distinct features. Elevated levels of IL-18 or IFN-gamma are specifically and most prominently observed in COVID-19 or influenza patients, respectively [79,80]. Findings in COVID-19 patients still need to be consolidated. However, there is strong evidence that a hallmark of severe COVID-19 infection is a delayed type I/III interferon response [81]. It remains to be fully understood which molecules play a critical role in disrupting the balance between viral clearance and collateral lung damage and are good correlates of disease severity. Serum levels may not accurately reflect the production of inflammatory molecules in the lower respiratory tract. Therefore, it is important to study the viral-induced immunopathology in the lung-specific microenvironment. COVID-19 has highlighted the major contribution of lung endothelial cells to pathogenesis [82]. There is growing evidence that lung epithelial and mesenchymal cells are also playing a regulatory role in the response to viral infections (reviewed in [83]). A challenge will be to further define the role of cell-to-cell heterogeneity within each cell type through single-cell studies [84,85]. Another challenge will be to identify and integrate the multiple cellular pathways that control the cellular response to viral infection. Interestingly, there is increasing evidence for a cross-talk between metabolism and hyperinflammation [86,87]. In line with these observations, obesity and diabetes are associated with a higher risk of developing a severe form of COVID-19 and influenza pneumonia [88–90].

Viral and host genetic determinants of the immune response remain largely unknown. Specific mechanisms evolved by highly pathogenic IAVs and CoVs to counteract the type I interferon response have been described [91,92]. Loss-of-function mutations in interferon induction or signaling genes, or the presence of autoantibodies with interferon neutralizing activity, predispose patients to severe COVID-19 [93,94] or severe influenza [95]. An initiative for genome-wide mapping of host genetic factors associated with COVID-19 was launched recently [96] and would deserve to be extended to influenza disease.

### 2.2. Elucidating the mechanisms for extrapulmonary tropism and/or complications

Although SARS-CoV-2 infection affects primarily the respiratory tract, many extrapulmonary dysfunctions have been reported in severe COVID-19 patients, especially in the central nervous system (CNS), kidney, liver, gastrointestinal tract, and cardiovascular system [97,98]. The most commonly reported extrapulmonary dysfunctions in influenza patients affect the CNS and the cardiovascular system [99]. A vast majority of published observations focus on emerging zoonotic (SARS-CoV, MERS-CoV, and highly pathogenic IAVs of the H5 or H7 subtypes) and pandemic (SARS-CoV-2 and H1N1pdm09) viruses. The extent to which infections with seasonal viruses can lead to extrapulmonary complications might be underappreciated. One of the most prominent extrapulmonary symptoms associated with SARS-CoV-2 is anosmia [100], a feature also observed during influenza virus infections, but to a lesser extent [101]. Olfactory dysfunctions are related to the tropism of both viruses for the olfactory epithelium. Infection of the olfactory epithelium induces direct (replication) or indirect (inflammation) damage to olfactory neurons, leading to anosmia [102,103]. In the case of long-term persistence of anosmia post-COVID-19, an anterograde propagation of the virus to the olfactory bulb and CNS is suspected [104]. Influenza-associated encephalopathies are reported with a relatively high incidence in Japan, which could be due to more reporting and/or to a genetic predisposition [99,105].

The mechanisms underlying extrapulmonary manifestations remain largely unknown. One of the proposed mechanisms is direct tissue damage caused by IAV or CoV infection as there is evidence that the viral receptors (sialic acids and ACE2, respectively) and transmembrane protease serine 2 (TMPRSS2) protease, which cleaves the surface glycoproteins (hemagglutinin and Spike, respectively), both required for viral entry, are expressed at extrapulmonary sites [106,107]. Immunohistochemistry detection of viral nucleic acids or antigens in extrapulmonary tissues has been reported on autopsy samples and in animal models [99,108,109]. However, the robustness and clinical relevance of these findings remains debatable. Moreover, there is no strong evidence for viremia in COVID-19 or influenza patients, so the mechanisms for extrapulmonary spread of infectious viruses, if any occurs, remain unclear. There is evidence for influenza virus CNS invasion via retrograde axonal transport in the olfactory nerve in an immunocompromised child [110] and in animal models [108]. Whether CoVs and in particular SARS-CoV-2 could use this route of neuroinvasion remains unclear [102,103,111].

Beyond direct viral toxicity, other proposed mechanisms for extrapulmonary pathology include dysregulation of the immune response and endothelium inflammation and damage [97,99,112]. It is important to determine to what extent viral replication, and possibly viral persistence, can occur outside the lung, to understand organ-specific pathophysiologies and to identify the viral and host (e.g., age, sex, comorbidities, and genetic traits) determinants involved. This knowledge is needed to improve therapeutic interventions not only during the acute phase of disease, but also in the longer term in patients that experience post-acute syndromes [113].

### 2.3. Tackling the burden of bacterial coinfections and rethinking antimicrobial stewardship

Bacterial coinfections and secondary infections are detected in severe influenza patients with a high frequency (11 to 35% in most studies) and are a major cause of morbidity and mortality [114]. Most reports indicate they are detected only in low proportions in severe COVID-19 patients [115–117], although there are contradictory observations [118]. For instance, the proportion was 2.3% in a large multicentre prospective cohort of patients hospitalized during the first wave of the pandemic in the United Kingdom [117]. Different biases may have led to underestimation of bacterial coinfections in COVID-19 patients, including the accelerated patient flow, lack of microbiological diagnosis, effect of barrier measures, and, possibly, high antibiotic use. Whether and how bacterial infections affect the outcome of COVID-19 remains unclear so far [117,119]. It is essential to extend the clinical and basic knowledge in this field, especially as there is evidence that treatments targeting immune and inflammatory responses (e.g., corticosteroids and IL-6 inhibitors) could increase the risk of bacterial superinfections [120,121].

The mechanistic understanding of how respiratory virus infections can favor bacterial superinfection needs to be improved. Multiple mechanisms have been described in the case of influenza infections, including facilitation of the attachment of bacteria to the bronchopulmonary epithelium, impairment of the respiratory ciliary function, and alterations of the innate immune responses (reviewed in [122,123]). Bacteria recovered in influenza and COVID-19 patients are mostly gram-positive and gram-negative, respectively, suggesting that distinct mechanisms could be involved. However, the lung barrier damage resulting from interferon-λ signaling upon viral infection was recently proposed to cause increased susceptibility to bacterial superinfections in both influenza virus and SARS-CoV-2-infected mice [124,125]. There is growing evidence for bidirectional interactions between respiratory viruses and bacteria and for a complex interplay with the microbiome and the immune system [122,126]. So far, the mechanisms of copathogenesis have been investigated mostly in animal models. They remain to be explored in humans and to be taken into consideration in the development of clinical practices.

A shared concern regarding management of influenza and COVID-19 infections is the overuse of antibiotics (e.g., [127,128]), which might contribute to the emergence of multiantibiotic-resistant strains. An important challenge is therefore to rethink antibiotic stewardship and guidelines, to promote more systematic, early and rapid microbiological diagnostic approaches on admission to hospital, and to consider less empirical and more tailored treatments for each patient presentation.

## 3. Virus- and host-targeted therapies, vaccine development

### 3.1. Developing novel host-directed therapies

De novo drug development is notoriously a slow, expensive, and uncertain process. Drug repurposing, i.e., using a drug that has been validated as toxicologically safe and approved for another indication, represents a potentially time- and cost-effective strategy, especially in the context of pandemic response. However, this strategy has so far not led to the identification of any effective prophylactic or therapeutic treatment upon emergence of SARS-CoV-2 [129], with the exception of dexamethasone [130] and IL6 receptor blockers [131]. Successful drug repurposing against emerging respiratory viruses will require a better exploration of the drugs’ pharmacological and biodistribution properties and their suitability for lung delivery [132]. Being able to reliably evaluate which molecules are likely or unlikely to treat respiratory infections within approved therapeutic windows would allow to screen more effectively a larger proportion of the pharmacopeia. Developing formulations and/or delivery procedures distinct from the approved ones would minimize but not totally abrogate the benefits of repurposing over de novo drug design.

Host-directed therapies, which target host proteins essential for the viral life cycle and/or pathogenesis instead of viral proteins, can in principle provide the advantage of broad-spectrum efficacy and reduced antiviral resistance and can rely on the repurposing of approved drugs [133]. Immunomodulatory therapies hold particular promises for treating severe cases of influenza disease or COVID-19, which are frequently associated with an excessive and/or imbalanced release of pro-inflammatory cytokines and chemokines [1,81,134]. Regarding immunomodulatory treatment of the cytokine storm in severe influenza, published data based on randomized controlled trials are limited, and the efficiency of immunomodulatory therapy is still under debate [134]. Various immunomodulatory treatments, including corticosteroids, interferons, antagonists of the IL-1 or -6 receptors, or Janus kinase inhibitors, have been assessed clinically in severe COVID-19 patients with contrasting results (reviewed in [135]). Immunomodulatory drugs can delay viral clearance if administered prematurely and can also affect the course of tissue repair [83]. Therefore, a major challenge is not only to identify the most relevant immunomodulatory pathways but also to establish the optimal timing of intervention during the course of the disease. Drugs having immunoregulatory as well as antiviral activities might show the strongest benefits.

The combination of host-directed and/or conventional antivirals is an additional avenue of research, as it can potentially result in synergistic effects and prevent antiviral resistance [136–138]. In a randomized clinical trial on hospitalized adults with COVID-19, the combination of baricitinib and remdesivir was found to reduce the recovery time and to limit serious adverse events [139]. However, drug combination therapy remains a challenging approach, as not only the drugs biological activity but also their pharmacokinetics, biodisponibility, and mode of delivery have to be addressed in order to optimize synergistic effects.

### 3.2. Leveraging cutting-edge technologies in structural biology and computational tools to develop highly potent antivirals

**Fig 2:**
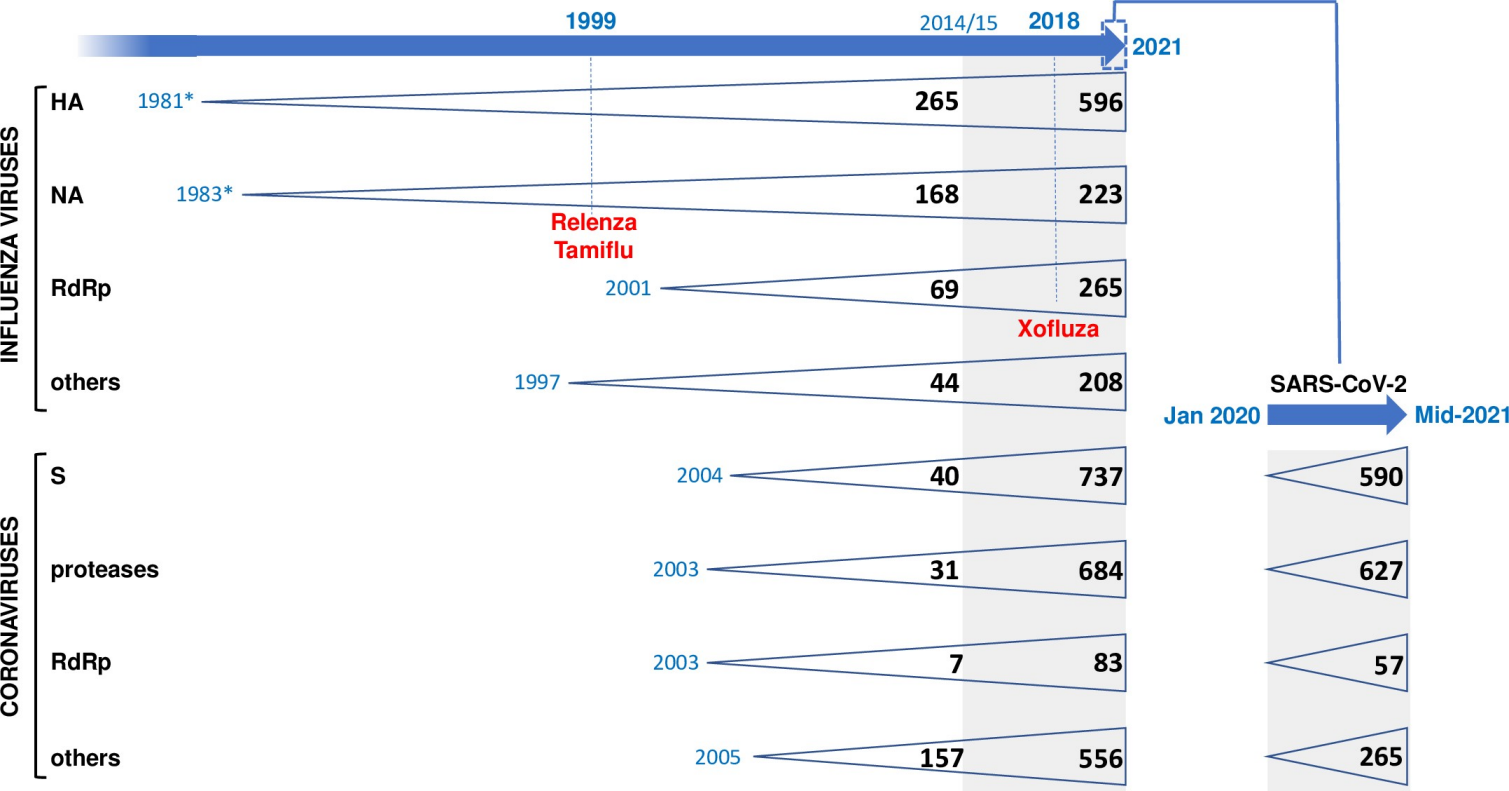
Growing pool of structural data available for influenza viruses and coronaviruses. The number of 3D structures available in the Protein Data Bank (https://www.rcsb.org, 17 October 2021) for each of the indicated category of viral proteins has been recorded separately for the 1981 to 2014 and the 2015 to 2021 period (the latter corresponding to an extended use of new generation detectors and cryo-EM, gray background). SARS-CoV-2 protein structures were counted separately (inset on the right). The years when the Relenza, Tamiflu and Xofluza inhibitors were approved by the FDA are indicated. RdRp, RNA-dependent RNA polymerase: PB1, PB2, and PA proteins of influenza viruses; nsp7, nsp8, nsp10, nsp12, and nsp14 proteins of corona viruses. Proteases: nsp3 and nsp5. * corresponds to the publication years of the first HA and NA X-ray structure, which preceded by several years their deposition in the PDB. HA, hemagglutinin; NA, neuraminidase; S, Spike; SARS-CoV-2, Severe Acute Respiratory Syndrome Coronavirus 2.

Structural biology plays a major role in drug development. Knowledge of the tridimensional (3D) structure of a target protein bound with a first ligand provides information that allows to design chemical modifications in order to improve the affinity of the ligand for the target and its biological activity, through iterative cycles of costructure determination/chemical optimization. Influenza was one of the first infectious diseases for which a rationale structure-based design of inhibitors led to the marketing of a drug [140]. More than 15 years of development were required between determination of the viral neuraminidase X-ray structures of IAV in 1983 and influenza B virus in 1992 [141,142] and the Food and Drug Administration (FDA) approval of the first neuraminidase inhibitors Relenza and Tamiflu in 1999 [143] (**Fig 2**). Almost 10 years separate the X-ray structure of the influenza polymerase endonuclease domain [144] and FDA approval of the endonuclease inhibitor Xofluza in 2018 [145]. A significant breakthrough occurred in the mid-2010s when new generation detectors enabled a tremendous expansion of the electron microscopy (EM) techniques. Cryo-EM does not depend on protein crystal formation and can unveil the flexibility inherent to the different conformational states of large complexes in the sample. As a result, the number of available 3D structures has been growing explosively, even for large or difficult-to-crystallize proteins such as membrane proteins: >600 structures of influenza virus proteins have been deposited in the Protein Data Bank between 2015 and mid-2021 and >1,200 structures of SARS-CoV-2 proteins in only 1.5 year (**Fig 2**). For instance, cryo-EM has allowed to visualize the conformational dynamics of IAV polymerase during the complete transcription cycle [146], IAV hemagglutinin along the process of membrane fusion [147], and SARS-CoV-2 spike protein at the surface of virions [148], thus increasing the mechanistic understanding of these proteins and opening new avenues for antiviral strategies. Cryo-EM was used to solve the structure of several SARS-CoV-2 proteins complexed with ligands (e.g., [149,150]). To date, X-ray crystallography and NMR still remain more suitable than cryo-EM for the high-throughput screening of small molecules libraries, an approach that was recently undertaken to identify inhibitors of SARS-CoV-2 main protease [151]. The field will benefit from further improvements of cryo-EM in terms of resolution and speed of data acquisition/processing and from the development of emerging structural biology and microscopy methods that allow to capture dynamic processes at the sub-nanoscale resolution and, for some, in native conditions [152,153].

Efforts to push further the current limits of structural biology techniques need to be backed up by advances in other disciplines, e.g., protein chemistry expertise for sample preparation, protein-structure prediction tools, and computational chemistry tools. The artificial intelligence program named AlphaFold represents a considerable leap in accurately predicting the 3D structure of proteins from their amino acid sequence [154]. Combined with extensive next generation sequencing, it will guide drug discovery and help understand the biological significance of amino acid variations, in particular in the context of a viral pandemic. Advanced computational and machine learning methods should also help improve the performance of in silico docking programs, whose limitations have been highlighted by the COVID-19 pandemic [155].

### 3.3. Developing improved vaccines with well-defined correlates of protection

Influenza vaccines have been administered each year since the 1940s to protect against seasonal influenza epidemics. Despite recent advances such as the development of live attenuated vaccines, current vaccines still show major limitations [156]. It has become a public health priority to develop next generation universal influenza vaccines capable to provide a more durable and broader protection, ideally against drifted seasonal viruses as well as against zoonotic or pandemic viruses of any subtype. Several approaches that target conserved regions of the virus such as the hemagglutinin stalk, or stimulate T cell–mediated immune responses, are currently in preclinical development [157,158]. Other strategies to improve vaccine effectiveness include a better understanding and forecasting of viral evolution, the optimization of neuraminidase content, the use of novel adjuvants, and the development of more efficient, nucleic acid–based, vector-based, or recombinant protein–based production platforms [158,159]. Computation- or structure-based design of the hemagglutinin antigens and nanoparticle display are being used to enchance vaccine immunogenicity [160,161]. The first Phase I clinical trial of a quadrivalent influenza nanoparticle vaccine candidate was launched by the National Institute of Health in June 2021 (https://bit.ly/2Ua0Lw3).

In contrast to influenza, no vaccine has ever been approved for the prevention of seasonal human CoV infection. However, vaccines are widely used to prevent CoV infections in domestic animal species such as cats, swine, cattle, and poultry, although their effectiveness is limited by a short duration of vaccine-induced immunity and by genetic drifting of the circulating viral strains [162]. Knowledge gained from CoV veterinary vaccines and the initial development of vaccines for SARS-CoV and MERS-CoV, combined with tremendous research efforts, led to the development, trial, and approval of several safe and effective vaccines against SARS-CoV-2 within 12 months [1,163]. Along with more traditional vaccine platforms, the mRNA vaccine platforms from BioNtech/Pfizer and Moderna, previously developed for cancer treatment and viral vaccines (e.g., Zika vaccine), were among the first to reach Phase I clinical trial and to be approved. mRNA vaccines now appear as a technology of choice in the context of pandemic response, owing to its simple and flexible manufacturing process and its safety profile [164]. Multiplexed chimeric spike mRNA vaccines could possibly offer broad-range protection against SARS-like CoV infection [165]. Several companies have started to develop an mRNA-based influenza vaccine. However, a number of challenges remain to be addressed. The determinants of mRNA vaccine efficacy and tolerability need to be better understood [166], while the cost-effectiveness of the manufacturing process and long-term storage stability still need to be improved [167].

For the ongoing development of universal influenza vaccines and improved COVID-19 vaccines, a key research area is the identification of good immune correlates of protection (CoP), which might then be used as a proxy of vaccine efficacy. The serum hemagglutination inhibition (HAI) antibody titer has been largely used as a CoP for influenza vaccines. However, its relevance and robustness to predict vaccine performance, especially for new universal vaccines, are subject to debate. Other potential immune CoP, such as CD8^+^ and CD4^+^ T-cell counts, interferon gamma-secreting cells counts, neuraminidase inhibition titers, nasal IgA or hemagglutinin-stalk antibodies titers, are currently under investigation [168,169]. In the case of SARS-CoV-2, epidemiological studies and studies of vaccine-induced immunity in nonhuman primates identified neutralizing antibodies as a CoP (e.g., [170,171]). The ongoing large-scale vaccination campaigns should allow the monitoring of multiple immune readouts and their analysis for CoP, which hopefully will facilitate the optimization of vaccine dose and schedule in the future. As with influenza viruses, a global monitoring of genetic and antigenic changes that occur in circulating SARS-CoV-2 viruses will be required to inform whether updating of the vaccine is required [172]. Finally, a number of important social issues, including vaccine hesitancy, vaccine equity, and access to vaccination in low-to-middle income countries, still remain to be tackled.

## Conclusions

**Fig 3:**
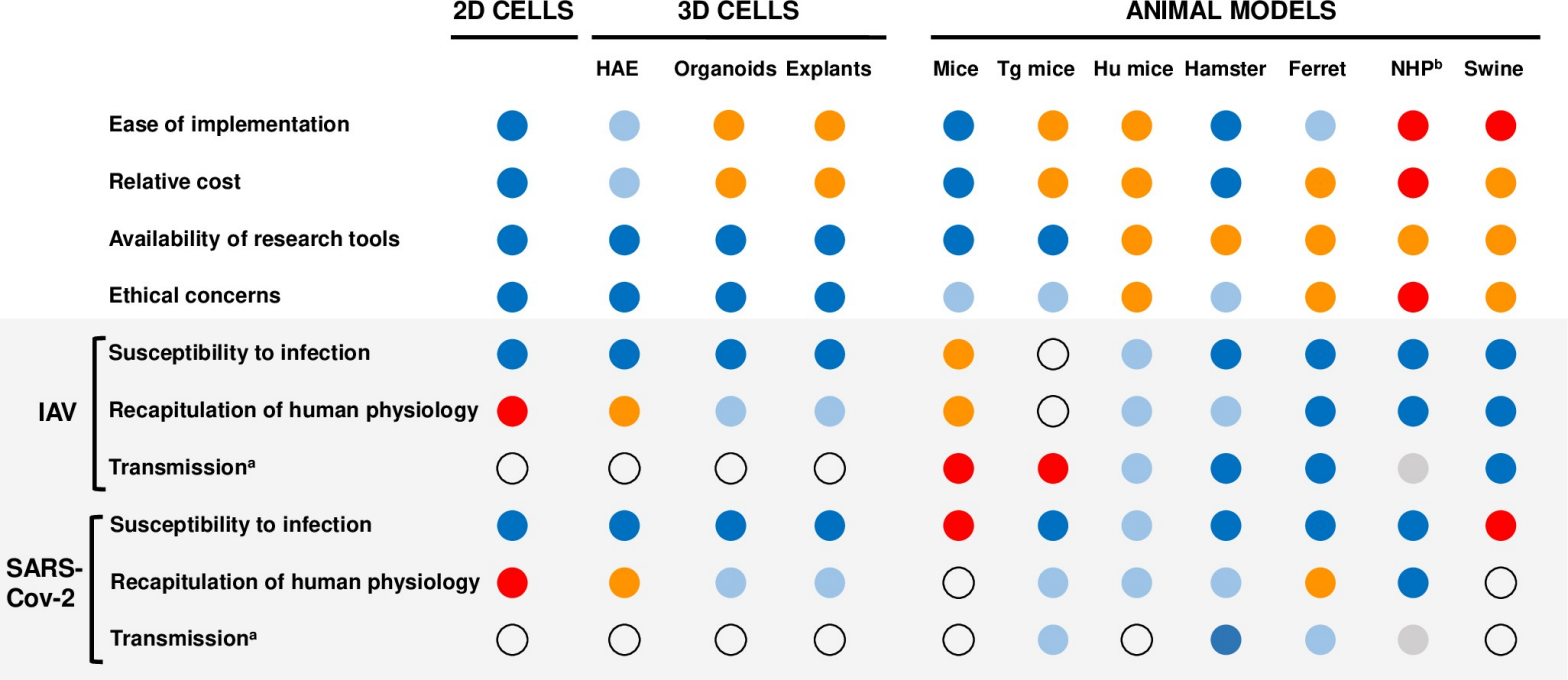
Cellular and animal models available for IAV and SARS-CoV-2 infections [174–176]. An estimation of the performance to the criteria indicated on the left is provided with the following color code: dark blue (very good), light blue (good), orange (poor), red (very poor), gray (not documented), no color (irrelevant). ^a^ All transmission routes including aerosol and direct contact routes. ^b^ NHP have been very rarely used in transmission studies; however, it was reported that H1N1pdm09 influenza viruses can efficiently transmit between marmosets [177]. 2D/3D, two- or three-dimensional; HAE, human airway epithelium; Hu mice, humanized mice (refers to mice grafted with human immune cells in the case of IAV and to mice grafted with human lung tissue in the case of SARS-CoV-2); IAV, influenza A virus; NHP, nonhuman primate; SARS-CoV-2, Severe Acute Respiratory Syndrome Coronavirus 2; Tg mice, transgenic mice.

Although influenza viruses and CoVs differ in many ways, several challenging research issues are common to both. It is very likely that synergies and cross-fertilizing ideas between influenza and CoV research will occur in the coming years. There is a “challenge in the challenge” for most of the topics addressed above: the development of physiologically relevant cellular and animal models (**Fig 3**). The cell lines that are widely used in influenza and CoV research (e.g., MDCK, Vero-E6, and A549) bear very little resemblance to the human respiratory epithelium, which can compromise the relevance and applicability of the findings. The use of primary cells, induced human pluripotent stem cells, and 3D lung-on-a-chip or organoid cultures should be facilitated and developed, to provide more accurate models for the differentiated respiratory epithelium or the microanatomy of the lung [173]. Likewise, it is crucial to set up animal models that mimic more closely the physiopathology in human patients and can provide reliable information for fundamental research as well as preclinical testing of therapeutic and vaccine candidates [106,174].

Each of the research topics mentioned above presents a breadth and level of complexity that call for synergies and multidisciplinary approaches. For instance, progress in understanding the complexity of airborne transmission will require a multidisciplinary approach that combines virology and clinical medicine with aerobiology, biophysics, chemistry, mathematical modeling, and engineering. Cooperation will also be required outside the field of life sciences, e.g., with regulators and manufacturers for the development of novel vaccines and with the socioeconomics and humanities fields when it comes to vaccine acceptance or to the impact of human behavior (such as deforestation, intensive farming, consumption and trade of wild animals, or global traveling) on the risk of zoonotic viral emergences.

Finally, the global research ecosystem needs to be strengthened by consolidating and connecting to each other existing networks of expertise and by promoting rapid and effective data sharing through the establishment of flexible platforms and rigorous guidelines.
